# Alerting, orienting, and executive control in intellectually gifted children

**DOI:** 10.1002/brb3.2148

**Published:** 2021-07-19

**Authors:** Alexandre Aubry, Béatrice Bourdin

**Affiliations:** ^1^ Laboratoire Lorrain de Psychologie et Neurosciences de la dynamique des comportements (2LPN, UR 7489) INSPE de Lorraine, Université de Lorraine Nancy France; ^2^ Centre de Recherche en Psychologie: Cognition Psychisme et Organisation (UR UPJV 7273) Université de Picardie Jules Verne Amiens France

**Keywords:** attention network test, attentional processes, executive control, intellectual giftedness

## Abstract

**Introduction:**

Intellectually gifted children have higher performance in many domains of attention than intellectually average children. However, these empirical findings are not consistent in the literature. Few studies investigated the characteristics of alerting, orienting, and executive control networks in intellectually gifted children. The aim of our study was to investigate their characteristics of attentional abilities compared to intellectually average children.

**Method:**

Fifty‐five intellectually gifted children (age range 8–14 years old) were compared to 55 intellectually average children (age range 8–14 years old) using the Attention Network Test (ANT) to assess these three attentional constructs.

**Results:**

Intellectually gifted children made fewer errors than intellectually average children in the processing of the ANT. In terms of attention network scores, they also outperformed intellectually average children in executive control only.

**Conclusion:**

Intellectually gifted children do not differ from intellectual average children in terms of the speed of processing in a speeded task such as ANT, but they stand out in terms of accuracy of processing. Intellectually gifted children have better ability to focus volitionally in order to solve a simple perceptual conflict than intellectually average children.

## Graphical abstract

The aim of this study was to investigate the characteristics of three attentional networks in intellectually gifted children. The findings show the intellectually gifted children have better ability to focus volitionally in order to solve a simple perceptual conflict than intellectually average children.

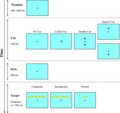

## 1 INTRODUCTION

Attention is related to intelligence (Burns et al., 2009; Schweizer et al., 2005). Intellectually gifted children (IGC) outperform in most attentional tasks (Johnson et al., 2003; Shi et al., 2013). However, this finding was not replicated in some studies (Montoya‐Arenas et al., 2018; Viana‐Sáenz et al., 2020, for a meta‐analysis). Some authors affirm IGC would have a higher prevalence rate of having attention deficit hyperactivity disorder (ADHD) than the general population (Chae et al., 2003; Karpinski et al., 2018; Rommelse et al., 2016, for a discussion). This prevalence may be overestimated, because IGC would have similar behaviors than ADHD (Alloway & Elsworth, 2012; Webb et al., 2005). High intelligence would be inherently related to characteristics indicative of ADHD such as inattention problems (Hartnett et al., 2004; Lee & Olenchak, 2015). Thus, these findings convey the idea that IGC may have lower quality of attention than that of intellectually average children (IAC). The reason of this inconsistent empirical findings would be related to some studies with severe selection bias (Karpinski et al., 2018, for instance) or methodological issues (Alloway & Elsworth, 2012, for instance). In addition, these characteristics of intellectual giftedness similar at those of ADHD are mostly observed in the clinical impressions than empirical findings (Rommelse et al., 2017). The aim of this current study is to investigate the characteristics of the attentional abilities in IGC.

IGC are more accurate and faster than IAC in the most of attentional tasks (Duan & Shi, 2014; Johnson et al., 2003). These empirical findings are not consistent. Some authors have shown only higher accuracy in the attentional tasks between IGC and IAC (Chae et al., 2003; Shi et al., 2013). In others words, IGC would have better control on their responses without having necessarily higher rapidity in the execution of the attentional tasks.

In simple elementary cognitive tasks (e.g., inspection time or choice reaction time tasks), IGC have faster response times than IAC (Duan et al., 2013; Kranzler et al., 1994). This discrepancy in response times may reflect a high degree of automatization processing in IGC (Gaultney et al., 1996). However, their rapidity of processing seems to be relative. The faster processing in IGC can be moderated by the demands of the cognitive task, to the extent that this difference between the intellectually gifted and average groups may disappear (Geary & Brown, 1991; Lajoie & Shore, 1986). In conclusion, high intelligence does not always reflect faster performance (Reams et al., 1990). In the same vein, some studies have shown an equivalent learning progression in intellectually gifted and average children (Vogelaar et al., 2017, 2019; Vogelaar et al., 2017). Although there are some contradictory results (Calero et al., 2011; Kanevsky & Geake, 2004), the rapidity of learning in IGC seems not to distinguish from IAC in terms of learning potential. The specificity of intellectual giftedness concerns the high capacity to learn and to generalize their newly acquired knowledge in other contexts (Calero et al., 2011; Vogelaar, Bakker, Elliott, et al., 2017; Vogelaar, Bakker, Hoogeveen, et al., 2017; Vogelaar et al., 2019). This high learning capacity might be linked to their high performance in reasoning (Caropreso & White, 1994), working memory (Calero et al., 2007; Hoard et al., 2008; Leikin et al., 2013; van Viersen et al., 2014), metacognitive abilities (Oppong et al., 2019), and executive functions (Arffa, 2007).

Reasoning, working memory, metacognitive abilities, and executive functions share some inter‐related processes such as cognitive control processes (Efklides, 2008; Engle, 2018; Fernandez‐Duque et al., 2000). These processes are an important step in attention, which is strongly related to intelligence (Burns et al., 2009; Cowan et al., 2006; Schweizer & Moosbrugger, 2004; Schweizer et al., 2005; Tillman et al., 2009; Tourva et al., 2015). IGC have higher performance than IAC in attentional tasks involving the cognitive control processes (Duan et al., 2009; Johnson et al., 2003; Liu et al., 2011a, 2011b). Furthermore, attention is a basic cognitive function essential for the deployment of high‐order cognitive processes. Attention is crucial to actively selecting aspects of our environment and to voluntarily regulating our cognitions and emotions (Posner et al., 2014). Attention allows the regulation of various neural networks through attentional networks involved in the alert state, orienting, or executive control (Posner & Rothbart, 2007).

The framework proposed by Posner and Petersen identified three attentional networks that are functionally and anatomically distinct: alerting, orienting, and executive control (Posner & Petersen, 1990; Petersen & Posner, 2012 for an update review). Alerting allows an optimal state of vigilance to be applied and maintained during a task (Petersen & Posner, 2012). The alerting network can be divided into two cognitive mechanisms: (a) tonic alerting is the capacity to self‐control alertness without any external cue, and (b) phasic alerting is the capacity to increase an individual's reaction after an external cue during a short period of time. Orienting is the ability to prioritize the processing of any stimulus by selecting salient information in a sensory input (Petersen & Posner, 2012; Posner, 1980). Executive control is the ability to focus volitionally in order to solve a simple conflict (Posner & DiGirolamo, 1998).

Some studies have investigated the relation of each attentional network to intellectual abilities. The phasic alerting effect seems not to be related to the IQ (Konrad et al., 2005; Tourva et al., 2016). However, the tonic alerting effect, which is one facet of the alerting network, is involved in intellectual performance (Schweizer & Moosbrugger, 2004; Schweizer et al., 2000). IGC have better attentional resources and are less inattentive than their peers for visual processing (Shi et al., 2013). However, their performance in attentional abilities seems to be influenced by the environment (family, school, and peers; Zhang et al., 2016). In the same vein, the orienting effect seems not to be linked to the IQ (Konrad et al., 2005; Tourva et al., 2016). Nevertheless, some studies have shown a relation between selective attention, that is the endogenous aspect of the orienting network, and intellectual abilities (Schweizer & Moosbrugger, 2004; Schweizer et al., 2005). The endogenous orienting refers to a voluntary process to disengaging, shifting, and re‐engaging the attention (Wainwright & Bryson, 2005). This facet of orienting networking involves a voluntary control of attention (Corbetta & Shulman, 2002; Posner et al., 2012; Rothbart et al., 2011). The control processes seem to be strongly related to intelligence. Some studies have shown that executive control plays an important role in intellectual functioning (Cowan et al., 2006; Engle et al., 1999; Engle, 2018). The executive control effect is significantly correlated with intellectual abilities (Konrad et al., 2005; Tourva et al., 2015). IGC show higher performances in executive control than nongifted children (Duan et al., 2009; Johnson et al., 2003; Oppong et al., 2019). However, others authors have not observed this high performance in executive control in IGC (Montoya‐Arenas et al., 2018; Viana‐Sáenz et al., 2020, for a meta‐analysis). There is thus a discrepancy in empirical findings on the particularity of executive control in IGC.

In summary, the empirical findings are inconsistent on the quality of attention in IGC. The behavior of IGC seems to be different in function of the type of attention assessed. To our knowledge, no study has investigated the difference between intellectually gifted and average children simultaneously in the alerting, orienting, and executive control networks. Based on the literature on typical development of attentional networks and its relationship with intelligence, we hypothesized that IGC will have better performance than IAC in only executive control.

## 2 METHOD

### 2.1 Participants

One hundred and eight French students were participated in this current study. The participants were in elementary and middle schools. The sample is composed of 54 IGC (mean age: 11.86 years, *SD*: 1.25, 22 females) and 54 IAC (mean age: 11.84 years, *SD*: 1.25, 22 females).

All typical children were recruited in elementary and middle schools. They had an IQ between 85 and 115, estimated using Raven's Standard Progressive Matrices (RSPM; Raven et al., 2000). All intellectually gifted children were recruited with the help of licensed psychologists or were enrolled in special gifted programs in middle school. The inclusion criterion for children in the intellectually gifted group was identified by the 95th percentile in one of two IQ tests such as the Wechsler Intelligence Scale for Children (WISC‐IV; Wechsler, 2005) and RSPM (Raven et al., 2000). Forty‐five IGC were identified by WISC‐IV and 9 gifted children by RSPM.

No children had known learning disorders; all children had normal or corrected vision, and all were native French speakers. The average of age is not dissimilar between the intellectually gifted and average groups, *t*(106) = 0.10, *p* = .92, *d *= 0.02 (Table 1). Both groups have an equal proportion of girls and boys, *χ* ^2^(1) = 0.00, *p* = 1.00, *φ* = 0.00. The information about our whole sample is summarized in Table 1.

**TABLE 1:** Description of the whole sample

Dependent variables	Intellectually gifted			Intellectually average		
	*M* (*SD*)	Skew	Kurtosis	*M* (*SD*)	Skew	Kurtosis
Overall						
RT (ms)	633.50 (86.09)	0.37	−0.67	641.78 (78.71)	0.11	−0.37
PE (% errors)	2.59 (1.91)	1.57	3.45	4.42 (3.49)	1.55	3.11
APE (Arcsin transformation)	0.15 (0.06)	−0.03	1.16	0.19 (0.09)	0.16	0.63
Alerting						
RT (ms)	27.31 (32.67)	−0.25	1.53	32.34 (30.95)	0.37	−0.23
PE (% errors)	0.02 (3.26)	−0.73	2.06	0.97 (3.11)	0.69	0.75
Combined z‐score	−0.58 (0.56)	−0.02	−0.10	−0.40 (0.46)	−0.13	−0.27
Orienting						
RT (ms)	46.18 (27.91)	−0.47	1.49	40.05 (34.51)	0.04	0.13
PE (% errors)	0.86 (2.41)	0.29	−0.14	1.02 (2.63)	0.43	−0.17
Combined z‐score	−0.24 (0.40)	0.00	1.47	−0.30 (0.57)	−0.08	−0.29
Executive control						
RT (ms)	83.70 (33.66)	−0.39	0.18	94.22 (32.46)	0.01	−0.32
PE (% errors)	3.80 (4.34)	1.22	0.90	5.94 (5.29)	1.08	1.80
Combined z‐score	0.57 (0.66)	0.38	0.02	0.95 (0.76)	0.20	0.14

Abbreviations: APE, Arcsin transformation of the Proportion of Errors; PE, Proportion of Errors; RT, Response Time.

### 2.2 Material

**FIGURE 1:**
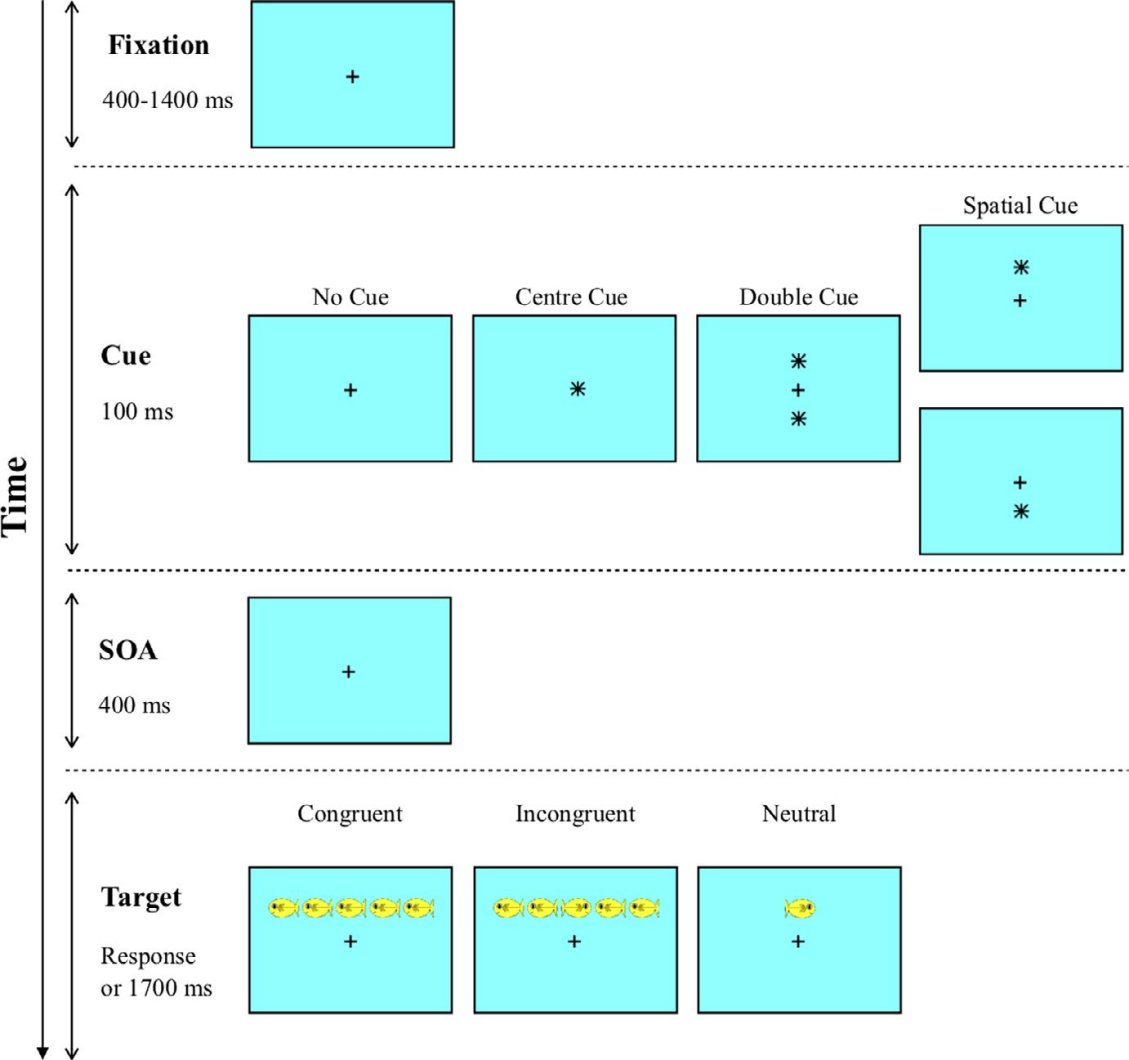
Procedure for the attention network test

The Attention Network Test–Child version (ANT; Rueda et al., 2004) was used to assess the performance in three independent attentional networks simultaneously: Alerting, orienting, and executive control. The ANT was computerized using PsychoPy (Peirce et al., 2019). All stimuli of the task were downloaded at this address: https://www.sacklerinstitute.org/cornell/assays_and_tools/ant/jin.fan/. The participant was instructed to indicate the direction of the central fish by pressing the left or right button on the keyboard, as rapidly as possible without making an error. As presented by Figure 1, each trial had the same structure: (a) a fixation cross was shown during a time period randomly fixed between 400 and 1,400 ms; (b) one of 4 cues was displayed on the screen for 100 ms: no asterisk was shown on the screen (no cue condition); an asterisk appeared in the middle of the screen instead of the fixation cross (central cue condition); an asterisk was displayed below and another above the fixation cross (double cue condition); or the correct location of the target below or above the fixation cross was indicated at 100% by an asterisk (spatial valid cue condition); (c) after a stimulus‐onset asynchrony (SOA) fixed at 400 ms, the target called flanker was displayed alone or flanked by four other stimuli on the screen: either a single fish above or below the fixation cross (i.e., neutral condition), or the middle fish in the same direction as 4 others (i.e., congruent condition), or the middle fish in the opposite direction to 4 others (i.e., incongruent condition); (d) the target was presented until the participant responded or for a duration limited to 1,700 ms. Then, the next trial started again at step 1. The task began with a training session using a block of 24 trials, including feedback for 500 ms. Then, the participants had to perform 288 trials without feedback divided into 3 experimental blocks of 96 trials each, with a 5s break between each block (Rueda et al., 2004, for similar procedure).

Following the recommendation of MacLeod et al., (2010), the present study considers both proportion of error (PE) and response time (RT) as performance measures. For each participant, average accuracy and median response times (RTs) were recorded for each condition. RTs were considered only for correct trials; RTs faster than 200 ms or slower than 1,700 ms were considered as extreme values and excluded from analyses (0.72% of trials).

Each subsystem (i.e., alerting, orienting and executive control) is isolable with Donders’ subtraction method (Donders, 1969). The difference score derives from a difference between a baseline measure and a related measure of interest. Thus, the difference in these two measures is assumed to be the additional stage of processing (Klein, 2003, for a detailed description). Consequently, we computed the effect of each attentional network based on this subtraction method from dependent variables (i.e., RT or PE) of each condition (Fan et al., 2002; see Rueda et al., 2004, for a similar scoring): (a) The tonic alerting effect was computed by subtracting no cue condition from double cue condition. The no cue condition imposes the participant to control them vigilance state for responding. The double cue condition only alerts the child to the imminent appearance of the target, without providing information about the target location. The subtraction of both conditions is able to estimate the alerting effect that is the effect of the target improving the alert state; (b) the orienting effect was computed by subtracting central cue condition to spatial valid condition. The central cue condition orients the attention to the screen center, whereas the spatial cue provides useful information about the target location. Participants have to disengage their attention from screen center and engage toward the future localization of the target. Thus, the difference between these two conditions indicates the child's ability to orientate their attention toward a new and salient stimulus (i.e., orienting effect); (c) the executive control effect was computed by subtracting incongruent condition from congruent condition. The incongruent condition requires the child to control their response in relation to the congruent condition. Thus, the difference between both conditions is able to estimate the participant's control ability to respond efficiently.

A composite attentional network score was computed by the combination of RT and PE differences scores into a single measure (Unsworth et al., 2020, for a similar procedure). These differences scores (i.e., RT and PE) of each attentional network were z‐scored. These *z*‐scores then were averaged. This form of integrating speed and error into a single score is a variant of the balanced integration score (Liesefeld et al., 2015; Liesefeld & Janczyk, 2019). This type of outcome has two advantages: (a) it makes easier to interpret the performance in each attentional network, and (b) it avoids the need to correct for multiple testing (Draheim et al., 2016; Hughes et al., 2013; Liesefeld & Janczyk, 2019). The interpretation of the composite attentional network score is simple: The higher composite score, the lower performance in the attentional network.

### 2.3 Procedure

In both child groups, all legal guardians received and signed an informed consent which exposed the aim of this current study. Before testing took place, the objectives of the study were explained to the children, who then gave oral assent for their participation. The present research was conducted in accordance with the ethical standards laid down in the Declaration of Helsinki. All participants performed the ANT task collectively within their group in around 20 min.

### 2.4 Data analysis

The data and R scripts required to replicate the statistical analyses are available on the Open Science Framework platform at https://osf.io/x4bpr/?view_only=cc363e6d273447009da3f10970b31bf1. All data analyses were conducted using R 4.0.3 (R Core Team, 2020). Following the recommendations of MacLeod and colleagues (2010), both RT and PE were analyzed separately. Both dependent variables were considered as performance indicators for attentional networks in terms of speed and accuracy (Wickelgren, 1977).

The permutation method used to compute the reliability of each attentional network score (in RT and PE; see MacLeod et al., 2010, for a detailed description). For each participant, all trials were randomly split into two halves 1,000 times. A correlation with a Spearman‐Brown's correction was computed for each split. For each attentional network score, the reliability coefficient is the average of the 1,000 corrected correlations (see Results section below).

The overall RT and PE were compared in both groups. For this, pairwise comparisons were performed. The violation of normality was assessed by skewness and kurtosis. The variance ratio was realized to estimate the violation of homogeneity variance (Blanca et al., 2018). All pairwise comparisons were realized with Welch's *t* test adjusted by a Holm's correction of *p*‐values (Holm, 1979). When the distribution of the overall RT or PE is skewed or the variance between both groups is unequal, Welch's *t* test is lower sensitive than Student's *t* test (Delacre et al., 2017; Rasch et al., 2011). All effect sizes were computed using Cohen's *d*.

The comparison of performance in both groups for each attention network was performed using repeated‐measures analyses of variance (RM‐ANOVA). For all repeated‐measures designs, the sphericity assumption was assessed by Mauchly's test (Mauchly, 1940). When this test revealed a violation of the sphericity assumption, a subsequent Greenhouse–Geisser correction was applied to the results of analysis. For all statistical analyses, the violation of each statistical assumption (i.e., normality and homoscedasticity) was checked using a graphical visualization. The influence of extreme values was assessed using Cook's *D* statistics (Cook, 1977). The cutoff of case identification was determined to use the *F* distribution with *df* = (*k* + 1, *n* – *k* − 1) and *α* = 0.50, where *k* is the number of predictors, and *n* is the number of observations (Cohen et al., 2003). In all statistical analyses, no Cook's D statistics exceed the threshold computed by the formula from Cohen et al., (2003). As recommended by Bakeman (2005), all effect sizes for mixed design were computed using generalized eta squared ($\eta_{G}^{2}$).

## 3 RESULTS

### 3.1 Comparison between intellectually gifted children and intellectually average children

#### 3.1.1 Overall response time and errors

We first investigated the group effect on the median RTs and the average of PE separately, regardless of the conditions. Table 1 shows the results for each group. An arcsin transformation has been applied on the PE in order to manage the violation of normality assumption of residuals (Unsworth et al., 2009 for similar problem).

The intellectually gifted group was not significantly faster than intellectually average group, *t*(105.16) = 0.52, *p* = .302, *d* = 0.10. However, IGC had significantly fewer errors than IAC, *t*(96.43) = 3.13, *p* = .002, *d* = 0.60.

#### 3.1.2 Attentional networks

**FIGURE 2:**
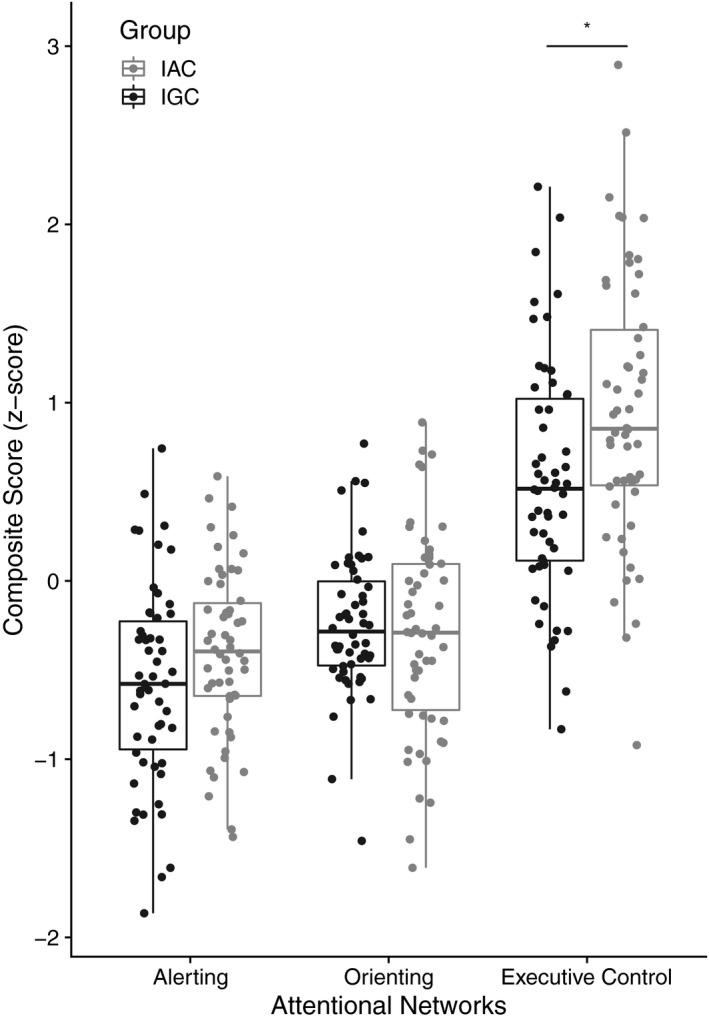
Performance at each attentional network by group

The executive control network score was most reliable, *r*_split‐half_ = 0.650, CI 95% [.644, 0.657], followed by the alerting network, *r*_split‐half_ = 0.204, CI 95% [.195, 0.212], and the orienting network, *r*_split‐half_ = 0.035, CI 95% [.026, 0.045]. The results related to networks indexes are illustrated for the three attentional networks in Figure 2.

A significant main group effect was found, *F*(1, 106) = 5.97, *p* = .016, $\eta_{G}^{2}$ = 0.02. The Tukey's HSD post hoc analysis showed IGC had lower attentional network indexes than IAC, *t*(106) = −2.44, *p* = .016. There were a significant interaction between group and attentional networks, *F*(1.84, 195.15) = 4.22, *p* = .019, $\eta_{G}^{2}$ = 0.02. The Tukey's HSD post hoc analysis with Holm's correction indicated that IGC performed better than IAC only in executive control network, *t*(314.58) = −3.45, *p* = .002. Both groups did not distinct on the alerting and orienting networks scores (*p*s > .05).

## 4 DISCUSSION

In the literature, some studies have suggested IGC would have inherent characteristics similar to ADHD, such as inattention problems (Alloway & Elsworth, 2012; Hartnett et al., 2004; Lee & Olenchak, 2015; Webb et al., 2005). Attentional difficulties were mostly observed by parents and teachers (Rommelse et al., 2017). In contrast, others authors have shown IGC outperform in most attentional tasks (Johnson et al., 2003; Shi et al., 2013). Attention is not an unitary construct (Chun et al., 2011; Petersen & Posner, 2012). Few studies have explored the different aspects of attention in the same intellectually gifted group. Some authors have shown IGC outperform in some domains of attention, such as tonic alerting (Shi et al., 2013) and executive control (Duan et al., 2009; Liu et al., 2011a, 2011b). However, these empirical findings have not been observed in others studies (Montoya‐Arenas et al., 2018; Viana‐Sáenz et al., 2020). The results of this current study provide additional information on the characteristics of the attentional abilities in IGC.

IGC did not differ from the IAC on the overall RT, but they were more accurate than the IAC for the ANT. The accuracy seems to be the key element of the high performance of IGC in the attentional tasks (Lajoie & Shore, 1986).

On the phasic alerting effect, our results highlighted that the performance of the IGC did not differ from that of the IAC. Their alertness would not be disparate from those of their peers when an external cue is given. Although IGC have better performance in tonic alerting (i.e., sustained attention) (Shi et al., 2013), our study has shown that IGC do not have higher performance than IAC in phasic alerting. The intelligence seem not be related to this aspect of the alerting (Konrad et al., 2005; Tourva et al., 2016). On the orienting effect, the IGC seems to have the same efficiency as the IAC. This finding confirms the independence between orienting network and intelligence (Konrad et al., 2005; Tourva et al., 2016). In the executive control effect, we have additional evidence that IGC have better performance than IAC (Calero et al., 2007; Duan et al., 2009; Johnson et al., 2003; Liu et al., 2011a, 2011b). Our results showed that the executive control effect is more efficient in IGC than their peers that is to focus volitionally in order to solve a simple perceptual conflict. In the literature, executive control is considered to be important in the functioning of working memory, namely capacity to process, manipulate, and briefly store information during a cognitive activity (Engle et al., 1999; Kane et al., 2001). This cognitive ability is strongly related to intellectual capacities (Ackerman et al., 2005). Some studies have highlighted that IGC have higher performance in working memory than IAC (Calero et al., 2007; Hoard et al., 2008; Leikin et al., 2013; van Viersen et al., 2014).

These results convey IGC do not outperform in all aspects of the attention. This could explain this divergence between parent's or teacher's impressions assessed by questionnaires and empirical findings on the attentional abilities in IGC (Rommelse et al., 2017). The exploration of the different aspects of attention therefore seems to be important to understand eventual difficulties in this area.

### 4.1 Limitations of the study

In our study, split‐half estimates for each network score demonstrate low reliability for the alerting and orienting networks and high reliability for the executive control network. This pattern of the reliability of network scores is classically found in the ANT (MacLeod et al., 2010). This weakness in the reliability of the network scores have to force us to interpret the results with caution, particularly about the alerting and orienting networks.

The child version of ANT was used to estimate the efficiency of each attentional network (Rueda et al., 2004). This task used only the spatially valid cue to assess the orienting effect. The use of invalid and valid cues can increase the orienting effect and improve the observation of the development of orienting networks with age (Abundis‐Gutiérrez et al., 2014; Waszak et al., 2010).

In the current study, the design of this version of the ANT could not investigate the interaction between the attentional networks. In the literature, some studies showed the presence of interactions between the attentional networks (Callejas et al., 2005; Pozuelos et al., 2014). These interactions modulate the efficiency of each attentional network, and the effect of this modulation is influenced by child development (Mullane et al., 2016; Pozuelos et al., 2014). A modified ANT was created to specifically investigate these interactions (Callejas et al., 2004, for details). In future studies, investigation into the interaction between networks may bring another perspective about the attentional development in IGC.

Despite being fun and game‐like, the child version of ANT becomes quickly boring for children (Ishigami & Klein, 2015), as well as for young adults. Boredom might affect the participant's engagement in the attentional task. Thus, the participant's performance could be underestimated in all conditions of this type of task. Some authors have shown that it is possible to make a game‐like tool for assessing attentional networks which is more engaging than the ANT (e.g., Klein et al., 2017).

## 5 CONCLUSION

The current study has investigated the different aspect of the attention in IGC (e.g., phasic alerting, orienting, and executive control networks). First, IGC are more accurate than fast on ANT compared to IAC. The accuracy seems to be an important characteristic of intellectual giftedness in the attentional tasks. Second, our finding indicates that IGC have only higher executive control performance than IAC. This result confirms that executive control is an important cognitive characteristic in IGC (Duan et al., 2009; Johnson et al., 2003). Executive control plays a crucial role in the functioning of the working memory, that is, the capacity to process, manipulate, and briefly store information in the current cognitive activity (Engle et al., 1999; Kane et al., 2001), and intellectual functioning (Cowan et al., 2006).

## CONFLICT OF INTEREST

The author(s) declared no potential conflicts of interest with respect to the research, authorship, and/or publication of this article.

## AUTHOR CONTRIBUTION

AA and BB designed the study. AA performed data collection and analyzed the data. AA and BB wrote the manuscript.

## ETHICAL STATEMENT

The study was carried out in accordance with the recommendations of French psychological research and the Declaration of Helsinki. All parents gave consent, and each child gave their written assent to be part of the research. We also had approval from headmasters and teachers.

### PEER REVIEW

The peer review history for this article is available at https://publons.com/publon/10.1002/brb3.2148.
